# Inhibition of Notch enhances efficacy of immune checkpoint blockade in triple-negative breast cancer

**DOI:** 10.1126/sciadv.ado8275

**Published:** 2024-10-30

**Authors:** Qiang Shen, Kiichi Murakami, Valentin Sotov, Marcus Butler, Pamela S. Ohashi, Michael Reedijk

**Affiliations:** ^1^ Ontario Cancer Institute, University Health Network, 610 University Avenue, Toronto, Ontario M5G 2M9, Canada.; ^2^ Department of Medical Oncology and Hematology, Princess Margaret Cancer Centre, University Health Network, Toronto, Ontario, Canada.; ^3^ Department of Medicine, Division of Medical Oncology, University of Toronto, Toronto, Ontario, Canada.; ^4^ Department of Immunology, University of Toronto, Medical Sciences Building, 1 King’s College Circle, Room 7205, Toronto, Ontario M5S 1A8, Canada.; ^5^ Department of Medical Biophysics, University of Toronto, Toronto Medical Discovery Tower, MaRS Centre, 101 College Street, Room 15-701, Toronto, Ontario M5G 2M9, Canada.; ^6^ Department of Surgical Oncology, Princess Margaret Cancer Centre, University Health Network, 610 University Avenue, Suite 8-411, Toronto, Ontario M5G 2M9, Canada.

## Abstract

Aberrant Notch, which is a defining feature of triple-negative breast cancer (TNBC) cells, regulates intercellular communication in the tumor immune microenvironment (TIME). This includes tumor-associated macrophage (TAM) recruitment through Notch-dependent cytokine secretion, contributing to an immunosuppressive TIME. Despite the low response rate of TNBC to immune checkpoint blockade (ICB), here, we report that inhibition of Notch-driven cytokine-mediated programs reduces TAMs and induces responsiveness to sequentially delivered ICB. This is characterized by the emergence of GrB^+^ cytotoxic T lymphocytes (CTLs) in the primary tumor. A more impressive effect of sequential treatment is observed in the lung where TAM depletion and increased CTLs are accompanied by near-complete abolition of metastases. This is due to (i) therapeutic reduction in Notch-dependent, prometastatic circulating factors released by the primary tumor, and (ii) elevated PD ligand 1 (PD-L1) in lung metastases, rendering them profoundly sensitive to ICB. These findings highlight the potential of combination cytokine inhibition and ICB as an immunotherapeutic strategy in TNBC.

## Teaser

Sequential cytokine and PD1 inhibition as therapy for triple-negative breast cancer.

## INTRODUCTION

Breast cancer is the most common cancer diagnosed, and the second leading cause of cancer death in North American women (*1*, *2*). While successful targeted therapies have been developed for the estrogen receptor and/or progesterone receptor expressing luminal A/B subtypes, and for the human epidermal growth factor receptor 2–amplified subtype, there is an unmet need for effective targeted treatment for breast cancers that lack the expression of these receptors [triple-negative breast cancer, (TNBC)], the clinical surrogate of basal-like breast cancer (BLBC) (*3*). Compared to other breast cancer subtypes, TNBC is responsible for disproportionate years of life lost because it is aggressive, it recurs early, and it affects young women in the prime of life (*4*). In the past decade, a concerted effort has been made toward identifying molecular therapeutic targets in this subtype, leading to the adoption of novel agents including poly(adenosine 5′-diphosphate–ribose) polymerase inhibitors, antibody-drug conjugates, and immune-checkpoint inhibitors (*5*). In addition to these targets, the Notch signaling pathway has been identified as a driver and potential therapeutic target in TNBC (*6*–*11*).

Notch is an evolutionarily conserved intercellular signaling system, which is essential to cell differentiation during embryonic development and postnatal life. In canonical Notch signaling, upon binding of Notch ligands, Notch receptors undergo a series of proteolytic cleavages that release Notch intracellular domain (N^IC^) from the cellular membrane. N^IC^ undergoes nuclear translocation and engages the DNA binding protein CBF1/Suppressor of Hairless/Lag-1 (CSL) and a multiprotein co-activator complex, initiating transcription of target genes. In the breast, Notch is involved in mammary stem cell maintenance, progenitor cell fate, and is essential for normal mammary gland development. Pathologic Notch activation is a defining feature in BLBC/TNBC (*11*–*13*), and our recent work shows that Notch promotes BLBC in part, by regulating the expression of interleukin-1β (IL-1β) and CC chemokine ligand 2 (CCL2) from malignant cells (*14*). These inflammatory cytokine mediators shape the tumor immune microenvironment (TIME) through the recruitment of tumor-associated macrophages (TAMs).

The tumor microenvironment (TME), where tumor cells dynamically interact with resident and recruited “nonmalignant” cells through a complex network of matrix remodeling enzymes, growth factors, and cytokines, is crucial to malignant progression and metastasis (*15*). In addition to angiogenic vascular endothelial cells, fibroblasts, and other “nonmalignant” stromal cells, the breast cancer TME is characterized by immune cells of both the innate and adaptive systems (*16*–*21*). The pattern of immune infiltration differs among breast cancer subtypes. Compared to less-aggressive breast cancer subtypes, TNBCs are more infiltrated by immune cells, and the pattern of immune infiltration is strongly associated with outcome. High TAM count is inversely related to survival (*22*, *23*), while high tumor-infiltrating lymphocyte (TIL) count, specifically cytotoxic T lymphocytes (CTLs) is associated with improved survival (*24*). Through complex cross-talk in the TME, TAMs can suppress immune surveillance, stimulate angiogenesis, and promote tumor cell migration and metastasis with the underlying mechanisms largely unknown (*23*, *25*, *26*).

Immunotherapies targeting immune checkpoint proteins [immune checkpoint blockade (ICB) therapy] such as programmed death 1 (PD1) receptor and ligand (PD-L1/2) represent a major breakthrough to reboot suppressed CTLs. Despite clinical success in some malignancies such as melanoma, non–small cell lung cancer, and renal cell carcinoma (*27*–*33*), ICB has only demonstrated modest activity in breast cancer. Recent phase 3 trials in TNBC that compared standard chemotherapy plus ICB to chemotherapy alone demonstrated improvements in complete pathologic response [64.8% versus 51.2%; KEYNOTE-522 (*34*)] and overall response [56.0% versus 45.9%; IMpassion130 (*35*)]. These promising, yet less-than-satisfactory results expose the need for additional immune priming strategies to improve ICB response rates in TNBC. Immunosuppressive TAMs (*36*–*38*), which can suppress CTLs through immune checkpoint–independent mechanisms (*39*–*42*), are an obvious therapeutic target through Notch inhibition, to boost a response to ICB.

In the present study, we show that by reshaping the TIME, inhibition of Notch or Notch-driven cytokine-mediated programs including CCL2 or IL-1β, reverses immunosuppression in TNBC, potentiating ICB. Sequencing Notch/cytokine inhibition followed by ICB is critical to reducing macrophage infiltration, increasing activated tumor-infiltrating CD8^+^ T cells and reducing primary tumor growth. PD-L1 signal and the response rate to ICB are heightened in the lung, resulting in near-complete elimination of metastases. These findings illuminate the potential of sequential treatment in primary and metastatic TNBC.

## RESULTS

### Inhibition of Notch reboots the sensitivity of TNBC to ICB

**Fig. 1.**
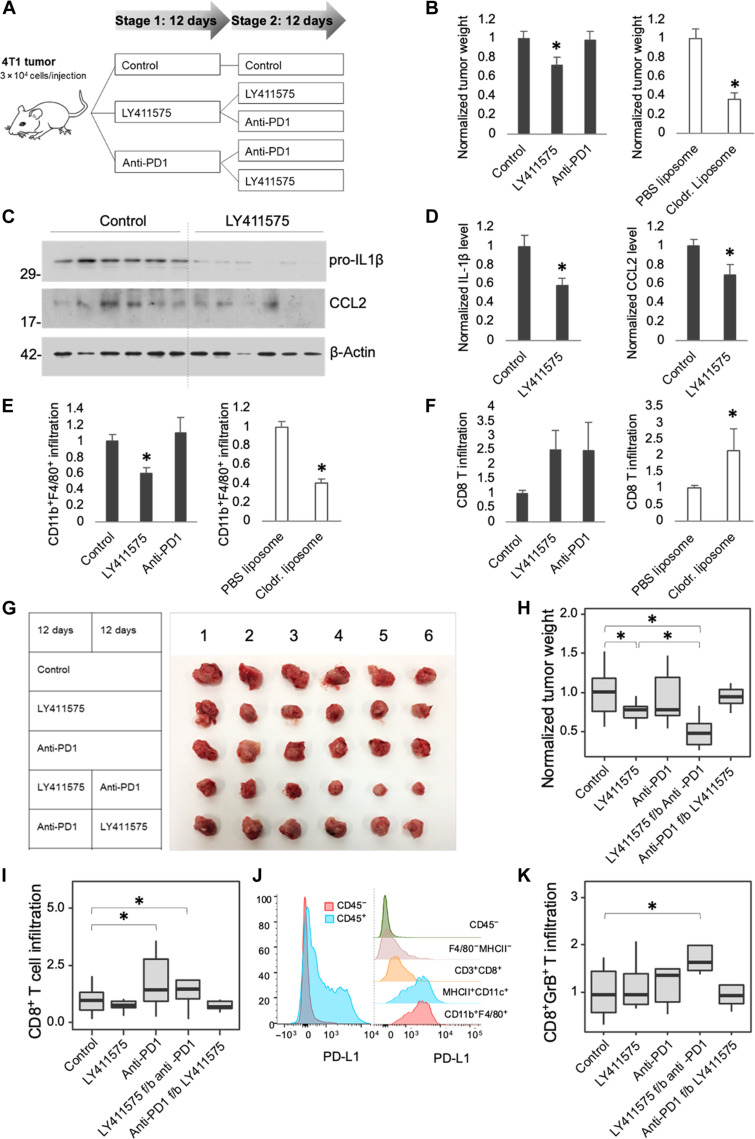
Inhibition of Notch resensitizes 4T1 mammary allografts to ICB. (**A**) Schematic diagram of the experimental design for two-stage crossover treatment of 4T1 tumor allografts, see text for details. (**B**) Tumor weight after stage 1 vehicle control, LY411575, or anti-PD1 treatment (left graph, *n* = 18 tumors per group) or PBS liposome or clodronate liposome (right graph, *n* ≥ 7 tumors per group). (**C**) Immunoblot of pro–IL-1β or CCL2 in tumor tissue after control or LY411575 treatment. Molecular weight markers are shown in kilodaltons. β-Actin is included as a loading control. (**D**) IL-1β and CCL2 levels in tumor tissue by enzyme-linked immunosorbent assay (ELISA) (*n* = 6 tumors per group). Flow cytometric analysis of F4/80^+^CD11b^+^ TAM (**E**) and CD8^+^ T cell (**F**) infiltrates after stage 1 (left graph, *n* ≥ 6 tumors per group) or after PBS liposome or clodronate liposome treatment (right graph, *n* = 4 tumors per group). (**G**) Excised tumors following stage 2 monotherapy or sequential treatment. (**H**) Tumor weight after stage 2 (*n* ≥ 14 tumors per group; f/b = followed by). (**I**) Flow cytometric analysis of CD8^+^ T cell infiltrates after stage 2 (*n* ≥ 17 tumors per group). (**J**) Flow cytometry histogram of PD-L1^+^ level according to cell type in a 4T1 allograft. (**K**) Flow cytometric analysis of CD8^+^GrB^+^ T cell infiltrates after stage 2 (*n* = 5 tumors per group). Bar graphs represent mean + SEM. Bloxplots represent median ± interquartile range (IQR), whiskers indicate 1.5 × IQR. *P < 0.05.

To examine the immune landscape and therapeutic response of TNBC to combined Notch-inhibition and ICB, we used an in vivo allograft model using murine basal-like mammary tumor 4T1 cells, as previously described (*14*). Tumor cells were orthotopically injected into the mammary fat pads of BALB/c mice. Using a crossover design, mice were randomly allocated to treatment with either the Notch pathway γ-secretase inhibitor (GSI; LY411575), anti-PD1 ICB (RMP1-14), or control treatment for 12 days (stage 1) followed by randomization to a second 12-day period (stage 2) with the same, or one of the other treatments (Fig. 1A). As a positive control for the effect of reducing macrophage infiltration in the TIME, clodronate liposome was administered (*43*). While both LY411575 and clodronate reduced primary tumor growth, no therapeutic effect was observed with anti-PD1 treatment at the end of stage 1, supporting previous clinical studies showing a low response rate to ICB in TNBC (Fig. 1B and fig. S1A) (*44*). The expression and secretion of the Notch targets, including IL-1β and CCL2 (*14*), were down-regulated after stage 1 (Fig. 1, C and D, and fig. S1, B to D). The reduction in tumor growth following LY411575 or clodronate was accompanied by a depletion of CD11b^+^F4/80^+^ TAMs (Fig. 1E), specifically the CD206^+^ M2-like, immunosuppressive and protumoral phenotype (*45*, *46*) (fig. S1E). Fitting with the reduction in immunosuppressive CD206^+^ TAMs, by the end of stage 1 there was a trend toward increased CD8^+^ T cell infiltration following LY411575 treatment (Fig. 1F), without an effect on CD4^+^ T cell infiltration (fig. S1F).

Following treatment crossover and the completion of 24 days of treatment (stage 2), while the effect of Notch inhibition with LY411575 monotherapy persisted (Fig. 1, G and H), a more substantial therapeutic effect was observed with sequential Notch inhibition followed by anti-PD1. No therapeutic effect was achieved with reverse sequential treatment (anti-PD1 followed by Notch inhibition). Anti-PD1 alone or Notch inhibition followed anti-PD1 increased CD8^+^ T cell infiltration (Fig. 1I). Although CD11b^+^F4/80^+^ TAMs represent the major source of PD-L1 signal in the TIME (Fig. 1J), anti-PD1 alone was insufficient to increase the activated, granzyme B (GrB)–positive CD8^+^ T cell subtype (Fig. 1K). Rather, Notch inhibition followed by anti-PD1 was required to increase activated CD8^+^ T cells, consistent with previous reports that TAM mechanisms other than PD-L1, suppress CD8^+^ T cell activity (*42*, *47*–*49*). Identifying CD8^+^ T cells as the critical mediators of the therapeutic response, the effect was lost in mice where CD8^+^ T cells were depleted during sequential LY411575 f/b anti-PD1 treatment (fig. S2). These findings suggest that Notch inhibition reduces TAM infiltration and switches the TIME from a “cold” immunosuppressive to “hot” immunopermissive phenotype, primed for ICB.

### Inhibition of IL-1β or CCL2 reverses immunosuppression in the TME and potentiates anti-PD1 treatment

**Fig. 2.**
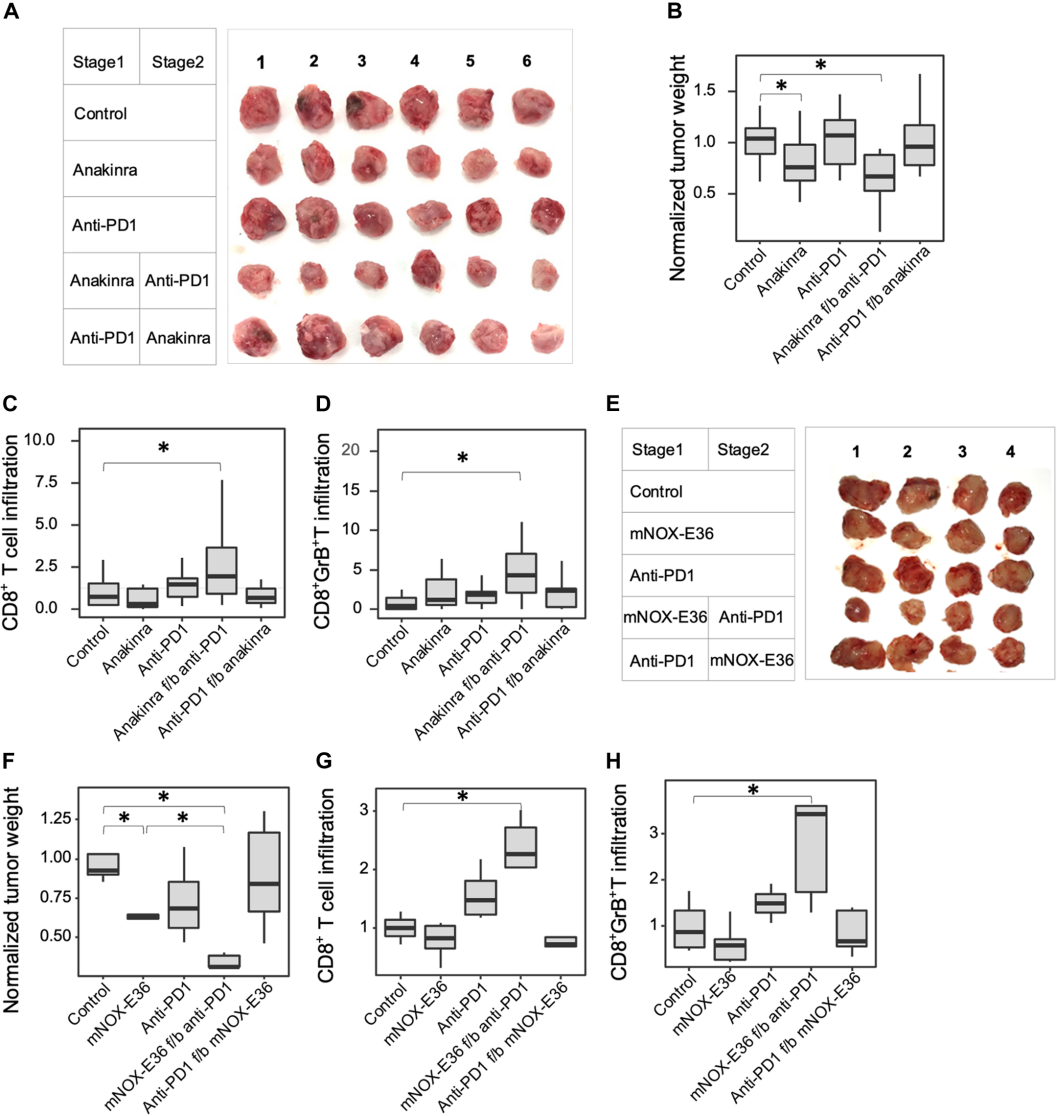
Inhibition of Notch-regulated cytokines potentiates ICB in 4T1 mammary allografts. (**A**) Excised tumors following stage 2 monotherapy (anakinra or anti-PD1) or sequential treatment. (**B**) Normalized tumor weight (*n* ≥ 12 tumors per group). (**C**) Flow cytometric analysis of CD8^+^ T cell infiltrates after stage 2 (*n* ≥ 10 tumors per group). (**D**) Flow cytometric analysis of CD8^+^GrB^+^ T cells after stage 2 (*n* ≥ 10 tumors per group). (**E**) Tumors excised following stage 2 monotherapy (mNOX-E36 or anti-PD1) or sequential treatment. (**F**) Normalized tumor weight (*n* ≥ 4 tumors per group). (**G**) Flow cytometric analysis of CD8^+^ T cells after stage 2 (*n* ≥ 4 tumors per group). (**H**) Flow cytometric analysis of CD8^+^GrB^+^ T cells after stage 2 (*n* ≥ 4 tumors per group). Bloxplots represent median ± IQR, whiskers indicate 1.5 × IQR. *P < 0.05.

Since Notch is ubiquitously expressed in normal tissues, systemic pan-Notch inhibition has been associated with undesirable side effects (*50*). Therefore, blocking Notch-regulated cytokines was considered as an alternative to combine with ICB. Anakinra is a recombinant IL-1β antagonist approved by the United States Food and Drug Administration (FDA) for the treatment of rheumatoid arthritis (*51*). Similar to the effects of LY411575, we found that tumor growth and CD11b^+^F4/80^+^ TAM infiltrates were reduced by anakinra treatment (Fig. 2, A and B, and fig. S3, A to C) with the greatest reduction in tumor growth achieved with anakinra followed by anti-PD1. Again, supporting that TAM depletion was a prerequisite, no therapeutic effect was observed with anti-PD1 monotherapy or with reversed sequential treatment. Delivering anakinra during both treatment stages combined with anti-PD1 at stage 2 offered no additional benefit, suggesting that IL-1β inhibition at stage 1 sufficiently primed the TIME (fig. S3, D and E). As observed for sequential LY411575 f/b anti-PD1 treatment, anakinra f/b anti-PD1 treatment converted the TIME to a “hot” phenotype, containing increased numbers of activated CD8^+^ T cells (Fig. 2, C and D).

NOX-E36 (emapticap pegol), a compound that targets CCL2 and has renoprotective effects in patients with macrophage-induced diabetic nephropathy (*52*), demonstrated similar findings (fig. S3, F and G, and Fig. 2, E to H). No therapeutic benefit was derived by combining anakinra and NOX-E36 ± anti-PD1, demonstrating that these cytokines were functionally nonredundant in this context (fig. S3, H to K). These findings reveal the therapeutic potential of pretreating TNBC with macrophage chemoattractant inhibitors, before ICB.

### Inhibition of Notch, cytokines, or PD1 reshapes the pulmonary immune microenvironment and reduces lung metastases

**Fig. 3.**
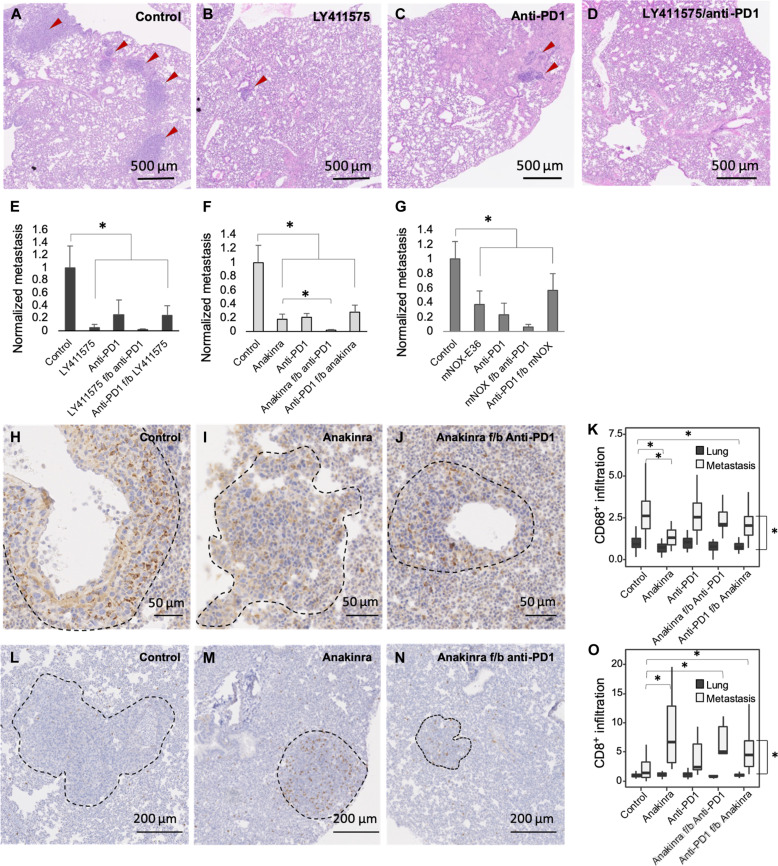
Inhibition of Notch, Notch-regulated cytokines, or PD1 reduces lung metastases. Representative histological images of metastatic foci in lungs of 4T1 tumor–bearing mice at the end of stage 2 treatment with (**A**) vehicle control, (**B**) LY411575, (**C**) anti-PD1, and (**D**) LY411575 f/b anti-PD1. (**E** to **G**) Normalized size of lung metastasis following monotherapy or sequential treatment (*n* = 3 mice per group). (**H** to **J**) Representative images of anti-CD68–stained lung after stage 2 of control, anakinra, or sequentially treated animals. (**K**) CD68^+^ infiltrates in metastases and adjacent lung following treatment (normalized to the number of CD68^+^ macrophages per mm^2^ of control lung; *n* ≥ 8 quantified regions from 3 mice per group). (**L** to **N**) Representative images of anti-CD8–stained lung after stage 2. (**O**) CD8^+^ infiltrates in metastases and adjacent lung following treatment (normalized to the number of CD8^+^ cells per mm^2^ of control lung; *n* ≥ 8 quantified regions from 3 mice per group). Bar graphs represent mean + SEM. Bloxplots represent median ± IQR, whiskers indicate 1.5 × IQR. *P < 0.05.

Breast cancer subtype is associated with specific patterns of metastases. Compared to other subtypes, TNBC display a higher tropism for lung (*53*). To study the effect of sequential treatment on TNBC metastases, lungs were excised and analyzed for metastases following stage 2 of the treatment regimen. All of the treatments that targeted Notch or its cytokine effectors reduced the size of lung metastases by at least 60%, which was greater than their effect on the primary tumor (Fig. 3, A to G). LY411575, which broadly regulates Notch-driven protumor programs in addition to cytokine secretion, including stem cell maintenance, proliferation, and invasion (*54*–*58*), reduced lung metastases by 95% compared to untreated, control animals (Fig. 3, B and E). In contrast to the lack of effect on primary tumors, anti-PD1 monotherapy reduced the growth of lung metastases by approximately 70%. When given sequentially following cytokine inhibition, anti-PD1 treatment further reduced lung metastases (Fig. 3, F and G).

Pursuing the clinical potential of sequential cytokine and PD1 inhibition further, the effect on lung immune infiltrates was tested. Tissue sections were stained with anti-CD68 (Fig. 3, H to J) or anti-CD8 (Fig. 3, L to N) antibodies, and immune infiltrates were quantified (Fig. 3, K and O, respectively), demonstrating immune cell predominance in the metastases compared to the adjacent lung. Mice receiving anakinra at the time of euthanasia had reduced macrophages in normal lung. A significant reduction of macrophages within metastases required 24 days of anakinra. Consistent with an immunosuppressive role for pulmonary macrophages, anakinra ± anti-PD1 resulted in elevated CD8^+^ T cell infiltrates within metastases. Confirming the anti-metastatic potential of CD8^+^ T cells, the therapeutic effect of Notch/PD1 inhibition was lost in mice where CD8^+^ T cells were depleted (fig. S4). The findings that emerge parallel those seen in the primary tumor and confirm the immunomodulatory effect and therapeutic potential of sequential cytokine and PD1 inhibition on the metastatic niche.

### Notch-dependent circulating tumor factors promote lung infiltration by macrophages and the growth of metastases

We hypothesized that the antimetastatic effect of Notch or cytokine inhibition may be through suppression of the primary tumor’s ability to condition distant metastatic sites. Randomized clinical studies comparing palliative systemic therapy (ST) to locoregional therapy (LRT) followed by ST in patients with de novo stage 4 breast cancer demonstrate an overall survival advantage when patients receive LRT, an evidence that the primary cancer can support the metastatic niche (*59*). Distinct cytokines are released from primary tumors, and the detection of these circulating factors can be prognostic, and inhibiting their activities can be therapeutic (*60*, *61*). To further profile Notch-dependent cytokines and their influence on metastases, serum cytokine arrays were performed. Serum from mice bearing 4T1 allografts treated either with or without LY411575 was compared to serum from tumor-free mice (fig. S5, A and B). Implicating Notch, several circulating cytokines, including IL-1β and CCL2, were increased >2-fold in mice with 4T1 tumors and were reduced >30% following LY411575 treatment [tissue inhibitor of metalloproteinase-1 (TIMP-1), stromal cell-derived factor-1 (SDF-1), IL-1β, granulocyte colony-stimulating factor (G-CSF), and CCL2]. LY411575-induced reduction of serum IL-1β and CCL2 was confirmed by enzyme-linked immunosorbent assay (ELISA; fig. S5, C and D).

To test whether Notch or cytokine inhibition influenced the pulmonary immune microenvironment before the onset of metastases, pulmonary immune infiltrates from 4T1 allograft–bearing mice were analyzed at the end of stage 1, a time point when lung metastases were not yet evident. Although there was no treatment effect on total CD45^+^ cell number (fig. S5E), CD11b^+^F4/80^+^ macrophage infiltration was reduced by Notch or cytokine inhibition (fig. S5F). To confirm the importance of tumoral Notch to this effect, mice bearing 4T1shN/J1 allografts in which Notch activity could be down-regulated by administering doxycycline were tested (*14*). Compared to wild-type mice and doxcycycline-treated 4T1shN/J1 allograft–bearing mice, untreated 4T1shN/J1 allograft–bearing mice demonstrated elevated CD11b^+^F4/80^+^ infiltrates (fig. S5G). These findings implicate tumoral Notch-driven cytokines in conditioning distant metastatic sites for macrophage uptake.

**Fig. 4.**
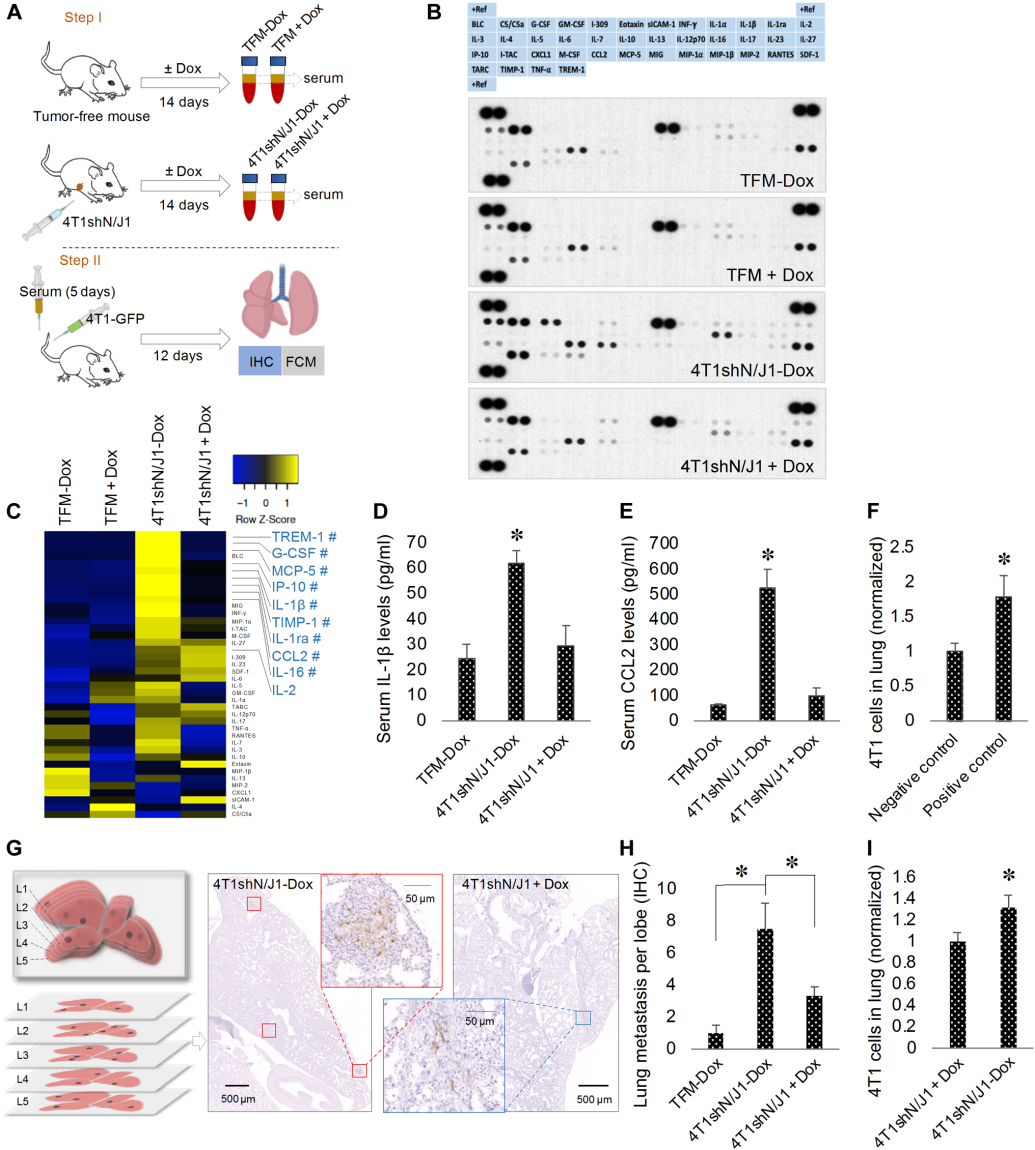
Notch activation in the primary tumor promotes lung metastases. (**A**) Schematic diagram of the experimental design: step I, serum is collected from TFM or mice bearing 4T1shN/J1 allografts (4T1shN/J1) treated with or without doxycycline (±Dox); step II, intravenous injection of serum from step I once per day for 5 days, followed by tail vein injection of 4T1-GFP cells into BALB/c mice and 12 days later, immunohistochemical (IHC) and flow cytometric (FCM) analysis of lung metastasis. (**B**) Cytokine arrays probed with serum collected in step I. (**C**) Heatmap of relative cytokine levels measured by array densitometry. The color key relates the heatmap colors to the standard score (*z* score). Cytokines, not influenced by Dox in TFM, increased >2-fold in 4T1shN/J1-Dox compared to TFM ± Dox serum are shown in blue. # indicates cytokines decreased more than 30% in 4T1shN/J1 + Dox. Serum levels of IL-1β (**D**) and CCL2 (**E**) by ELISA (*n* = 3 mice per group). (**F**) Flow cytometric analysis of 4T1 cells in lungs from 4T1 tail vein metastases models of untreated mice (negative control) or mice pretreated with intravenous rmIL-1β and rmCCL2 (positive control) (*n* = 4 mice per group). (**G**) Schematic diagram showing five 100-μm interval lung sections studied by IHC (left). Representative images of anti-GFP–stained sections of lungs from mice after step II (right). (**H**) Quantification of lung metastasis from mice after step II. All values are expressed as number of metastatic loci per lobe per mouse (*n* ≥ 7 mice per group). (**I**) Flow cytometric analysis of 4T1 cells in lungs from mice after step II (*n* = 10 mice per group). Data are presented as mean + SEM. *P < 0.05.

Pulmonary macrophages facilitate tumor cell extravasation and the establishment of metastasis (*62*). In vitro, this is supported by the finding that bone marrow–derived M2-like macrophages promote extravasation of 4T1 cells through BALB/c mouse lung microvascular endothelium (fig. S6, A and B). To prove that metastatic dissemination is promoted by Notch in the primary tumor, we collected serum from mice bearing 4T1shN/J1 allografts (Fig. 4A, step I). The serum contained a tumor- and Notch-dependent set of cytokines that overlapped with the circulating LY411575-dependent group [triggering receptor expressed on myeloid cells-1 (TREM-1), G-CSF, monocyte chemoattractant protein-5 (MCP-5), IP-10, IL-1β, TIMP-1, IL-1ra, CCL2, and IL-16; Fig. 4, B and C]. The Notch-dependent cytokines IL-1β and CCL2 were confirmed by ELISA (Fig. 4, D and E). Supporting the importance of Notch-dependent cytokines as mediators of metastases, conditioning the serum of mice with recombinant CCL2 (15 ng/kg) and IL-1β (1.2 ng/kg) at concentrations equivalent to those found in mice bearing 4T1shN/J1 allografts increased the formation of lung metastases (Fig. 4F). Compared to serum from untreated 4T1shN/J1 allograft–bearing mice, intravenous injection of recipient BALB/c mice with serum from doxycycline-treated 4T1shN/J1-bearing mice demonstrated reduced lung metastases in a 4T1 tail vein metastases model (Fig. 4, A, step II, and G to I). Thus, targeting Notch in the primary tumor or its cytokine effectors, may render the metastatic niche less potent, reducing disease progression.

### PD-L1 is up-regulated at the metastatic site

**Fig. 5.**
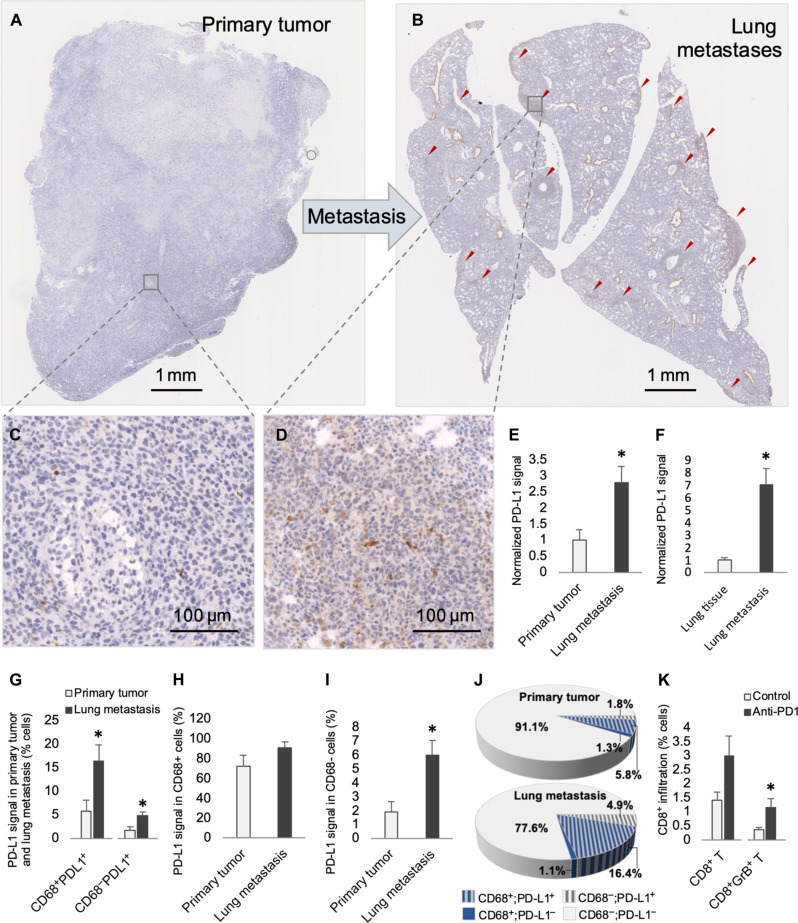
PD-L1 signal is up-regulated in 4T1 lung metastasis. Representative images of anti–PD-L1–stained sections of primary tumor (**A** and **C**) and paired lung (**B** and **D**) following stage 2, vehicle control. (**E**) Normalized levels of PD-L1^+^ cells in primary tumor and paired lung metastases (*n* = 4 mice per group). (**F**) Normalized levels of PD-L1^+^ cells in lung metastasis and surrounding lung tissues (*n* = 4 mice per group). (**G**) Percent PD-L1^+^ cells according to CD68 status in primary tumor and paired lung metastasis (*n* = 4 mice per group). (**H**) Percent PD-L1^+^ cells in the CD68^+^ fraction in primary tumor and paired lung metastasis (*n* = 4 mice per group). (**I**) Percent PD-L1^+^ cells in the CD68^−^ fraction in primary tumor and paired lung metastasis (*n* = 4 mice per group). (**J**) Graphical representation of percent cells according to PD-L1 and CD68 status from data shown in (G) to (I). (**K**) Percent CD8^+^ or CD8^+^GrB^+^ T cells in lung metastases following stage 2, vehicle control or anti-PD1 treatment (*n* = 6 mice per group). Data are presented as mean + SEM. *P < 0.05.

To investigate the robust response of lung metastases to ICB monotherapy, we tested PD-L1 levels at the metastatic site. Compared to the PD-L1 signal in primary 4T1 allografts, lung metastasis demonstrated approximately threefold higher PD-L1 signal (Fig. 5, A to E), and lung metastases demonstrated higher PD-L1 signal than surrounding normal lung (Fig. 5F). The threefold increase of PD-L1^+^ cells in metastases occurred in both the CD68^+^ and CD68^−^ compartments (Fig. 5G). The fraction of PD-L1^+^ cells in the CD68^+^ compartment was similar in the primary and metastases, indicating that increased PD-L1 in metastases was due to increased CD68^+^ macrophage infiltration, whereas in the CD68^−^ compartment, there was a proportional increase in PD-L1^+^ cells (Fig. 5, H to J). We speculated that elevated PD-L1–mediated immune suppression may render lung metastases more sensitive to ICB monotherapy. In contrast to primary allografts where combination therapy was required (Figs. 1K and 2, D and H), anti-PD1 alone increased activated CD8^+^ T cells in metastases (Fig. 5K).

## DISCUSSION

**Fig. 6.**
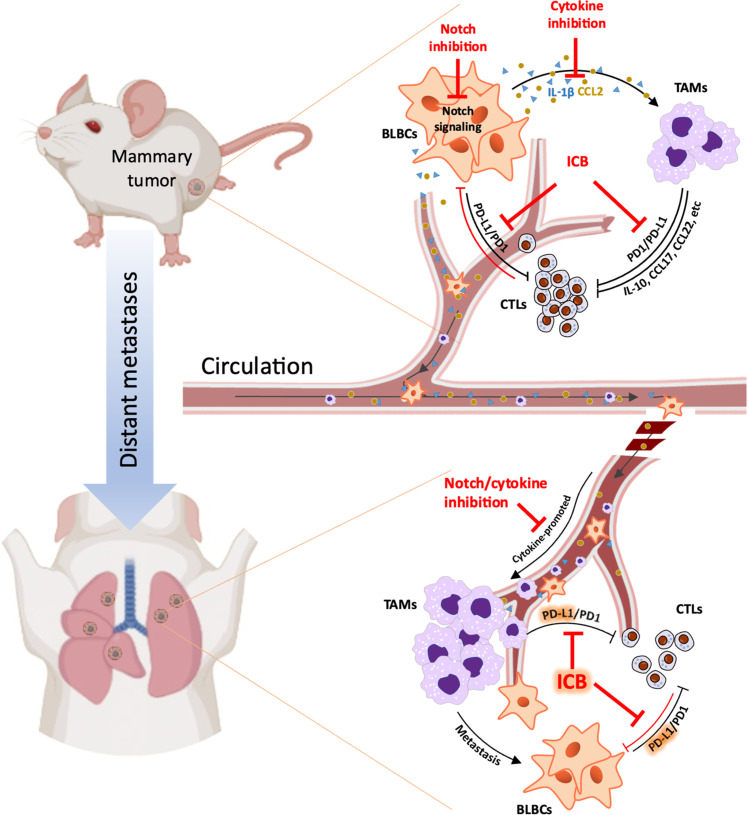
Inhibition of Notch or its cytokine effectors induces antitumor immunity and improves the efficacy of ICB at primary and metastatic sites of TNBC. See text for details.

The establishment of Notch as a driver in breast cancer and other malignancies has generated enthusiasm to therapeutically target this pathway or its downstream effectors. In addition to its autonomous oncogenic effects on tumor cells (*11*, *63*), Notch promotes tumor progression by regulating nonmalignant cells in the TME (*14*, *64*, *65*). This is mediated through juxtacrine signaling and also paracrine signaling through matrix remodeling enzymes, growth factors, and cytokines (*14*, *63*, *66*, *67*). We have shown that Notch shapes the tumor immunophenotype in TNBC through a vicious cycle of cytokine-mediated recruitment of TAMs and TAM-induced activation of Notch in tumor cells (*14*). Here, we showed that genetic or pharmacologic inhibition of Notch or the Notch-regulated cytokines CCL2 or IL-1β reduces TAM infiltration and reverses immunosuppression, potentiating ICB. While Notch or cytokine inhibitors impede disease progression, blocking PD1 substantially improves their therapeutic effect (Fig. 6).

Breast cancer is characterized by an inflammatory TIME that consists of immune cells of both the innate and adaptive systems. The qualitative, quantitative, and spatial patterns of immune infiltration vary between breast cancer subtypes. TNBCs can be stratified into several spatial immunophenotypes including “stroma restricted” or “fully inflamed” indicating respectively, the location of CD8^+^ infiltrates within stroma only or within both stroma and tumor cell clusters. “Poorly infiltrated” characterizes the absence of CD8^+^ infiltrates together (*68*). Compared to other subtypes, TNBCs are more likely to be immune cell-enriched, have high immune gene expression, and high expression of immunosuppressive genes (*69*). This includes the expression of PD-L1, which is crucial for immune self-tolerance, and a target for ICB (*70*). TNBCs that are immune-enriched and fully inflamed are associated with improved outcome, likely because of the juxtaposition of cytotoxic CD8^+^ T cells to malignant cells (*24*, *68*, *71*, *72*). This assumption placed high expectations on the clinical efficacy of ICB, which in practice were not realized (*44*). Consistent with findings in humans, we found anti-PD1 monotherapy to have a limited effect on primary 4T1 allografts and that TAMs were an impediment to its efficacy. While TAMs represent the predominant source of PD-L1 in 4T1 tumors, they also suppress CD8^+^ T cell number, mobility, and cytotoxicity through other mechanisms including IL-10–, TGF-β−, and prostaglandin-E2–mediated immunosuppression, or through depletion of L-arginine in the TME (*42*, *47*–*49*, *71*). In summary, immunosuppressive TAMs may be responsible for ICB failure in some cases, making them an important therapeutic target in tumors where they are abundant.

The aim of combination immunotherapy is to synergistically target tumor-promoting immunosuppressive programs. The requirement for a correctly sequenced treatment regimen depends on the mechanism of cancer immune escape. We have demonstrated that ICB-induced accumulation of activated GrB^+^ CD8^+^ T cells and maximal tumor killing requires first the elimination of immunosuppressive TAMs. Proving the importance of treatment delivery sequence, reverse sequential treatment provided no benefit. Prolonged targeting of TAMs, which are immunosuppressive through both immune checkpoint-dependent and checkpoint-independent mechanisms, produced a marginal therapeutic effect. However, the presence of other PD-L1–expressing cells in the tumor milieu likely blunt the response to anti-TAM therapy alone and explain the benefit of sequential ICB. The critical role of CD8^+^ T cells was verified by confirming that their depletion abrogated the effect of sequential treatment. This preclinical work provides the rationale for future clinical trials to test whether IL-1β− or CCL2-targeted therapy improves outcome in patients with stage 2/3 TNBC receiving standard-of-care (*34*) pembrolizumab ICB/taxane-platinum/anthracycline-based chemotherapy.

There are several considerations in selecting an anti-TAM therapy. IL-1β has traditionally been regarded as a product of immune cells and hence, preclinical models of IL-1β inhibition have not considered the key role of tumoral IL-1β (*73*, *74*). Our findings implicate tumoral Notch-driven programs in conditioning distant metastatic sites making Notch an obvious therapeutic target. Strategies have been developed that target each step of canonical Notch activation including GSIs and antibodies or inhibitors that target Notch receptors, ligands, and the Notch-mediated transcription complex (*75*). However, Notch activity is ubiquitous and predictably, first-in-human trials with first-generation GSIs identified substantial on-target side effects, including debilitating diarrhea resulting from secretory cell metaplasia of the intestinal epithelium (*76*). Nevertheless, when used at lower doses, decreased frequency, or in combination with corticosteroids or other supportive therapy, GSIs can be clinically effective and have an acceptable side effects profile. Owing to positive results in the recent phase 3 DeFi trial, nirogacestat has received FDA approval for adult patients with progressing desmoid tumors requiring systemic treatment (*77*). CB-103, which selectively inhibits N^IC^, was successful in a recent phase 1 study in patients with advanced adenoid cystic carcinoma, demonstrating an acceptable safety profile and limited antitumor activity (*78*). These encouraging clinical studies create on opportunity to combine next-generation Notch inhibitors with ICB. Clinical trial design and correlative studies will have to take into account that Notch regulates TAMs, lymphocytes, and cancer-associated fibroblasts in a context-dependent manner, with potential tumor-promoting effects (*55*).

Targeting cytokines directly with anakinra or mNOX-E3 may reduce side effects resulting from global Notch suppression and functions independent of the cytokine source. Anakinra and other IL-1 signaling antagonists including canakinumab and rilonacept, prescribed for inflammatory conditions, have a remarkable safety record (*79*) and experimental evidence, across most cancer types, supports a tumor-promoting role for IL-1β (*80*–*83*). In addition to recruiting TAMs and other protumoral inflammatory cells to the tumor, roles for IL-1β in growth, invasion, angiogenesis (*84*), metastases (*85*), stemness, and epithelial-to-mesenchymal transition (*86*) have been extensively described. Consequently, IL-1β up-regulation is generally associated with poorer prognosis in cancer (*87*). There is contrary evidence from a limited number of studies that IL-1β can have tumor-inhibiting effects, specifically by inducing both T helper cell 1 (Th1) and Th17 anti-tumorigenic effects in myeloma and lymphoma (*88*). However, the best available clinical information, derived from the phase 3 Cantos trial, which tested canakinumab to treat heart failure in a cohort of over 10,000 patients, showed that targeting IL-1β is associated with a reduction of death from all cancers (*83*), making it an excellent target for TAM depletion in combination with ICB.

Primary and metastatic breast cancers are immunologically distinct (*89*). TNBC metastatic lesions, characterized by lower TIL and elevated TAM counts, are more inert than their primary tumor counterparts, likely reflecting immune escape in cells that have colonized distant sites (*90*). In human TNBC lung metastases, immune cells demonstrate preservation of PD-L1 expression (*89*), providing an important layer of protection from immune surveillance. TAMs in 4T1 metastases express PD-L1 and, consistent with the successful IMpassion130 trial in humans with metastatic TNBC (*35*), expose a vulnerability to ICB. In our models, Notch-dependent circulating factors from the primary tumor facilitate deployment of TAMs to the lung parenchyma and the growth of metastases. Addressing the mechanisms of both TAM recruitment and suppression of local cytotoxic immune response (Fig. 6), sequential IL-1β inhibition followed by anti-PD1 provided a greater reduction in lung metastasis than either treatment alone, further supporting the addition of IL-1β antagonists to standard-of-care ICB-based ST in TNBC.

In summary, this study shows that Notch-driven cytokine programs promote TNBC progression, metastases, and anti-PD1 resistance, making this cancer subtype an ideal candidate for cytokine blockade in combination with ICB. If successful, this therapeutic approach could be used in other malignancies where macrophages are a key barrier to therapeutic efficacy.

## MATERIALS AND METHODS

### Reagents

LY411575 (A4019) was from APExBio (Houston, TX). InVivoMAb anti-mouse PD-1 (clone RMP1-14) antibody and InVivoMAb rat immunoglobulin G2a isotype control were purchased from BioXCell (Lebanon, NH). Anakinra (Kineret) was obtained from SOBI. mNOX-E36 was provided by NOXXON Pharma. Clodronate liposome and phosphate-buffered saline (PBS) liposome were from Encapsula NanoSciences (Brentwood, TN). IL-1β (H-153), CCL2/MCP-1 (R-17), β-actin, and horseradish peroxidase (HRP)–conjugated secondary antibodies were purchased from Santa Cruz Biotechnology. HES1 (D6P2U) antibody was from Cell Signaling Technology. CD8^+^ T cell depletion antibody was obtained in-house using the CD8 hybridoma cells (YTS169). Mouse IL-1β and CCL2/MCP-1 ELISA kits were from R&D Systems. Recombinant mouse CCL2 (rmCCL2), M-CSF (rmM-CSF), IL-1β (rmIL-1β), IL-4 (rmIL-4), and IL-13 (rmIL-13) were purchased from R&D Systems. SYBR green polymerase chain reaction (PCR) master mix, RPMI 1640 medium, Dulbecco’s modified Eagle’s medium (DMEM)/F12 medium, fetal bovine serum, penicillin-streptomycin, Matrigel basement membrane matrix (growth factor reduced), and calcein AM were from Thermo Fisher Scientific. Protease and phosphatase inhibitor cocktails were purchased from Roche. The RNeasyPlus Mini Kit was from Qiagen. iScript cDNA synthesis and DC protein assay kits were from Bio-Rad Laboratories. The Proteome Profiler Mouse Cytokine Array Kit (Panel A, ARY006) was purchased from R&D Systems. The Ferangi Blue Chromogen Kit 2 was from Biocare Medical.

### Mouse 4T1 mammary tumorgraft model

Eight-week-old female BALB/c mice were purchased from the Jackson Laboratory. Murine basal-like mammary tumor cells 4T1 (CRL-2539) were obtained from American Type Culture Collection and were orthotopically injected (3 × 10^4^ cells in 30 μl of PBS) into the mammary fat pad as previously described (*14*). Two days after injection and before tumors were palpable, mice were then randomly allocated to different treatment groups of Notch inhibition (LY411575; oral gavage at the dose of 5 mg/kg; daily), anti-PD1 ICB (RMP1-14; intraperitoneally at the dose of 12.5 mg/kg; every 3 days), anakinra (Kineret; IL-1 inhibitor; subcutaneously at the dose of 50 mg/kg; daily), mNOX-E36 (CCL2 inhibitor; subcutaneously at the dose of 20 mg/kg; every 2 days), clodronate liposome (intraperitoneally at the dose of 100 μl/10 g; every 3 days), or control treatments for 12 days (i.e., stage 1). Mice in each group were then randomly assigned to continue treatment, switched to another treatment, or combined with another treatment for a further 12 days (i.e., stage 2). In vivo depletion of CD8^+^ T cells was conducted by intravenous infusion via tail vein of YTS169 antibodies as previously described (*91*). At the end of each stage of treatment, primary tumor size (greatest diameter) and weight, tumor-infiltrating immune cells, and lung metastasis were analyzed. All animal experiments in this study (AUP #2994) were approved by the University Health Network Animal Care and Use Committee.

### Immunoblots

Tumor tissue samples were homogenized in RIPA [25 mM tris (pH 7.6), 150 mM NaCl, 5 mM EDTA, 1% NP-40, 1% sodium deoxycholate, and 0.1% SDS] lysis buffer freshly supplemented with protease and phosphatase inhibitor cocktails. After the insoluble components were pelleted at 15,000*g* for 3 min, the concentration of proteins in the supernatant was determined using the DC Protein Assay Kit. Equal amounts of proteins were then separated by 4 to 15% gradient gels. Proteins were then transferred onto polyvinylidene difluoride membranes (Bio-Rad) and blotted with corresponding primary antibodies. Following washing and incubation with HRP-conjugated secondary antibodies, the proteins of interest were visualized in HyBlot CL films (Denville Scientific) using ECL prime Western Blotting Detection Reagents (GE Healthcare). The films were then scanned and quantified using ImageJ software for protein levels.

### ELISA assay

IL-1β and CCL2 in tumor tissues were released by homogenizing the excised tissue samples, followed by a freeze-thaw cycle and centrifugation for 10 min at 4°C as previous described (*14*). Mouse serum samples were also collected following treatments as described. Cytokine levels in tissue lysates and serums were then analyzed using R&D Quantikine ELISA kits according to the manufacturer’s instructions.

### Flow cytometry

Tumors or lung tissues were minced and incubated in digestion buffer [collagenase (1 mg/ml) and pulmozyme (10 μg/ml), 2 mM l-glutamine, penicillin (100 U/ml), and streptomycin (100 μg/ml) in Iscove’s modified Dulbecco’s medium (IMDM)] at 37°C for 30 to 60 min. The digested samples were filtered through 70-μm Falcon cell strainers and the total viable cells were determined. Murine immune cells stained with fixable viability dye and antibodies targeting CD3, CD4, CD8, CD11b, CD11c, CD45, CD206, F4/80, GrB, MHCII, and PD-L1 (eBioscience) were analyzed with a BD Biosciences LSR Fortessa Analyzer using FlowJo software (TreeStar). Results are presented by relative values by comparing number of cells per gram of tumor tissues in treatment groups to that in control group.

### Quantitative real-time PCR

Total RNA was extracted using the RNeasyPlus Mini Kit according to the manufacturer’s protocol. cDNA was prepared from 1 μg of RNA using the iScript cDNA synthesis kit and subjected to quantitative real-time PCR using the default PCR cycle on a 7900HT Fast Real-Time PCR System (Applied Biosystems). Amplified DNA products were detected and quantified by SYBR Green using Power SYBR Green PCR Master Mix. Each sample was tested in triplicate for each primer set. Dissociation curve analysis was also performed to ensure the absence of nonspecific amplification.

### Immunohistochemistry

Formalin-fixed, paraffin-embedded mouse mammary tumors and lung tissues were cut into 4-μm tissue sections. As specifically indicated, some tissues were sectioned at several levels with 100-μm intervals. Then, all the sections were dewaxed and underwent heat-active antigen retrieval, followed by incubation with primary antibody targeting mouse CD8 (ab209775), CD68 (ab125212), green fluorescent protein (GFP; NB100-1678), or PD-L1 (D5V3B) overnight at 4°C. Secondary antibody and ABC reagent (Vector Laboratories) were added sequentially, and sections were developed with DAB reagent (Vector Laboratories) and counterstained with hematoxylin. Stained slides were scanned on a whole-slide scanner (Nanozoomer 2.0-HT, Hamamatsu, Japan). Sizes of the regions of interest were measured using NDP.view2 software (Hamamatsu) and further quantification of immunohistochemistry stained signals was done by using ImageJ software (National Institutes of Health) (*14*). Double staining of PD-L1 and CD68 was performed by first labeling PD-L1, followed by secondary antibody and ABC reagent. After the antibodies were stripped in denature buffer [25 mM glycine-HCl and 10% SDS (pH 2)] with DAB precipitate remained, the sections were sequentially processed with anti-CD68 antibody, secondary anti-rabbit-AP MARCH1 (BioCare), and Ferangi Blue Chromogen. Double staining of CD8 and GrB was performed by first labeling CD8 with DAB used as chromogen. After antibodies were stripped in denature buffer, the sections were then processed with anti-GrB antibody (AF1865), secondary antibody (MP-7401), and HIGHDEF yellow immunohistochemical (IHC) chromogen. The stained slides were scanned using the Vectra multispectral imaging system version 3 (PerkinElmer). The captured images were then unmixed using the Inform 2.4 Advanced Image Analysis software (PerkinElmer), which was further used to define the tumor compartment and trained to build algorithm (tissue segmentation, cell segmentation, phenotyping tool, and positivity score). After the algorithm was applied to batch analysis of all the images, the levels of infiltrated CD68^+/−^ PD-L1^+/−^ cells and CD8^+/−^ GrB^+/−^ cells were quantified.

### Serum cytokine Array

After mice were euthanized, blood samples were allowed to clot for 30 min at room temperature. Serum samples were then collected after centrifuging for 15 min at 1500*g* and examined for the major cytokines with a proteome profiler mouse cytokine array kit (Panel A, ARY006, R&D systems) according to the manufacturer’s protocol. In brief, the reconstituted mouse cytokine detection antibody cocktail was mixed with serum samples, which were then added onto the membranes precoated with capture antibodies and incubated overnight. After 3× wash with wash buffer and incubation with streptavidin-HRP, followed by 3× wash and addition of Chemi Reagent Mix, the immunoblotted cytokines were captured and visualized in HyBlot CL films. The films were then scanned and quantified using ImageJ software for the relative cytokine levels, which were processed with Heatmapper to visualize their pixel density as heatmaps.

### Bone marrow macrophage isolation and differentiation

Eight-week-old female BALB/c mice were used for isolation of mouse bone marrow–derived macrophages (BMDMs). In brief, after the mice were euthanized and the femurs were dissected, bone marrow cells were extruded from cavitas medullaris with DMEM/F12, followed by centrifugation for 10 min at 500*g*. The cells were then resuspended and cultured in DMEM/F12-10 supplemented with rmM-CSF (100 U/ml) and 1× penicillin-streptomycin in 10-cm polystyrene tissue culture dishes (Corning). On day 3, another 5 ml of freshly prepared medium was added. After 7 days, BMDMs were ready for further induction to M2 polarization (BMDM2) with rmIL-4 (10 ng/ml) and rmIL-13 (10 ng/ml).

### Tumor cell extravasation assay

BALB/c mouse primary lung microvascular endothelial cells were obtained from Cell Biologics and were seeded on basement membrane matrix-coated upper sides of transwell inserts (Corning Costar; 6.5-mm diameter and 8-μm pore size) and grown to confluent endothelial monolayers. The luminal and subendothelial (abluminal) compartments were thus generated in the upper and bottom chambers, respectively. Further, to examine whether infiltrated macrophages facilitate tumor cell extravasation, BMDM2 cells were preseeded in the bottom abluminal side of transwell inserts. Then, 500 μl of RPMI 1640 medium containing 10% fetal bovine serum was added to the bottom chamber and 200 μl of RPMI 1640 containing calcein-stained 4T1 cells was added to the upper chamber, and cells were incubated for 16 hours. After incubation, the transwell membranes were fixed with 3.7% formaldehyde for 10 min, followed by washing with PBS three times. The cells adhering to the upper side of the membrane were removed with a cotton swab, and transmigrated cells at the bottom side of the membrane were visualized using a fluorescent microscope and quantified as previously described (*92*).

### Blood collection and serum preparation

At the terminal point of 4T1 tumorgrafts with or without Notch inhibition LY411575 as previously described, mice were euthanized and serums were collected for serum cytokine assay. Serums from tumor-free mice were used as controls. In general, around 400 μl of whole blood could be collected per mice through cardiac puncture. Blood samples were allowed to clot for 30 min. Serum samples were then collected after centrifuging for 15 min at 1500*g*. Further, the tumorgrafts were repeated with 4T1 shN/J1 cells, which express doxycycline-inducible shRNAs that target murine NOTCH3 and JAG1 (*14*). Therefore, Notch signaling can be specifically knocked down in tumor cells by doxycycline administration. Serums were then collected, using serums from tumor-free mice with or without doxycycline as controls.

### Lung metastasis assay

Eight-week-old female BALB/c mice were first challenged by intravenously injecting the serums collected from tumor-grafted or tumor-free mice following different treatments as above described (100 μl/day) for 5 continuous days. For injection of rmIL-1β and rmCCL2, the daily dosages = (serum concentration differences between tumor-bearing mice and healthy mice) × 58.5 ml/kg (total blood volume) × 0.55. 4T1-GFP cells, which were generated by infection by GFP lentiviral plasmid (Santa Cruz Biotechnology) and selection by puromycin, were prepared at 5 × 10^5^ in 100 μl of PBS and injected into the tail vein and the mice were closely monitored every day for 12 days. Then, after the mice were euthanized, lung tissues were excised and processed for flowcytometric and IHC analysis.

### Statistics

Differences between two groups were evaluated using Student’s *t* test. Data are presented as the mean + SEM. For multiple comparisons, linear regression models were fit to test the null hypothesis that the treatment groups were equal to the control group. Initial results indicated heterogeneity in the variance for different treatment groups and that outliers were present in some samples. To minimize the impact of outliers, all models were fit using robust regression using iterated re-weighted least squares and robust standard errors were estimated using the HC3 estimator. Statistical analysis was performed using the R statistical programming language, the rlm and sandwich packages were used for modelling fitting and standard error estimation, respectively. Values of *P* < 0.05 were considered statistically significant.
