# Supplementary material for: Inhibition of Notch enhances efficacy of immune checkpoint blockade in triple-negative breast cancer
Source: Sci Adv. 2024 Oct 30;10(44):eado8275. doi: 10.1126/sciadv.ado8275 (PMC11524187; doi:10.1126/sciadv.ado8275)
Supplement: Supplementary file 1 — Figs. S1 to S6 [file sciadv.ado8275_sm.pdf]

Science Advances
AAAS

Supplementary Materials for

Inhibition of Notch enhances efficacy of immune checkpoint blockade in triple-negative breast cancer

Qiang Shen *et al.*

Corresponding author: Michael Reedijk, michael.reedijk@uhn.on.ca

Sci. Adv. **10**, eado8275 (2024)
DOI: 10.1126/sciadv.ado8275

This PDF file includes:

Figs. S1 to S6

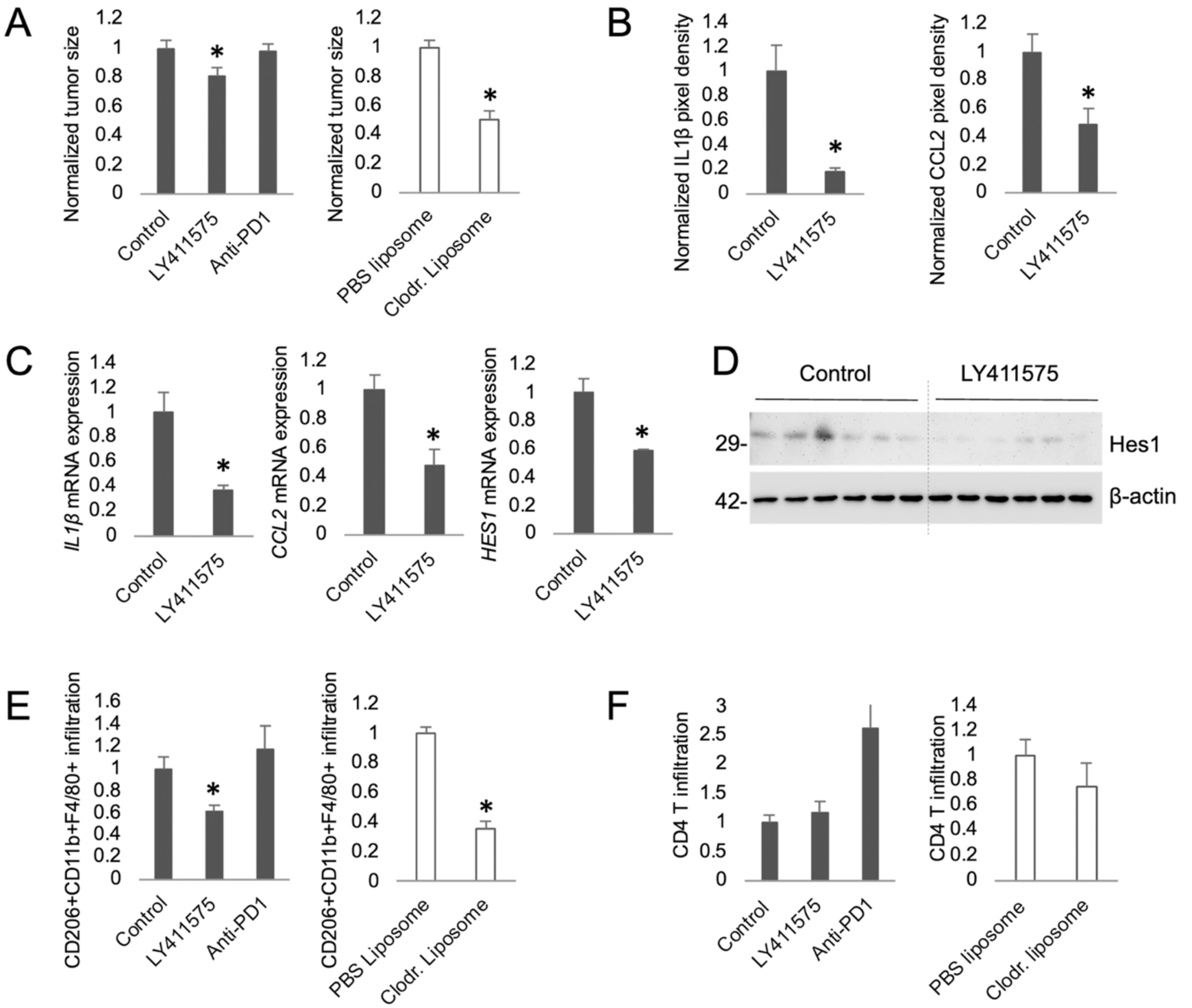

Fig. S1. Inhibition of Notch reduces CD206+ M2-like TAMs in 4T1 mammary allografts. A, Tumor size after stage 1 control, LY411575, or anti-PD1 treatment (left graph, n = 18 tumors/group) or PBS liposome or clodronate liposome (right graph, n ≥ 7 tumors/group). **B**, Expression of pro-IL1β and CCL2 from immunoblot in Fig. 1C determined by densitometry and normalized according to β-actin. **C**, *IL1β*, *CCL2*, and *HES1* mRNA expression in tumor tissue after control or LY411575 treatment (n ≥ 3/group). **D**, Immunoblot of Hes1 in tumor tissue after control or LY411575 treatment. Molecular weight markers are shown in kilodaltons. β-actin is included as a loading control. Flow cytometric analysis of CD206+CD11b+F4/80+ M2-like TAMs (**E**) or CD4+ T cells (**F**) after

stage 1 (left graph, $n \geq 6$ tumors/group) or PBS liposome or clodronate liposome (right graph, $n = 4$ tumors/group). Data are presented as mean + SEM. *, $P < 0.05$.

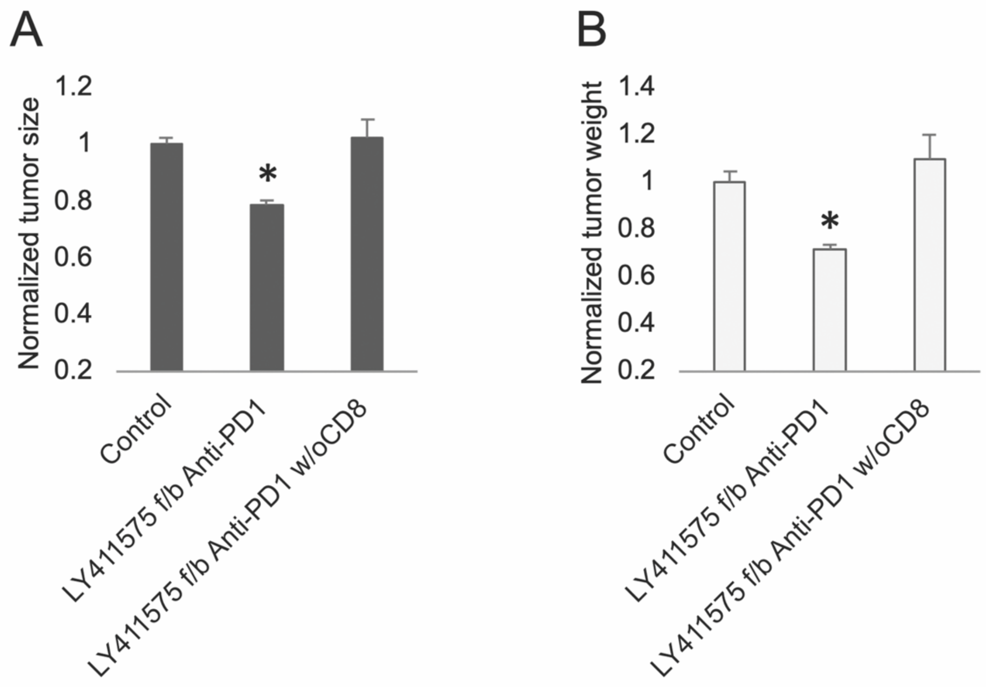

Fig. S2. CD8+ T cells mediate the therapeutic response to sequential Notch and immune checkpoint inhibition. Tumor size (**A**) and weight (**B**) after stage 2 control and LY411575 followed by anti-PD1 treatments in BALB/c mice, or LY411575 followed by anti-PD1 treatment in CD8+ T cell-depleted BALB/c mice (n = 12 tumors/group). Data are presented as mean + SEM. *, P < 0.05.

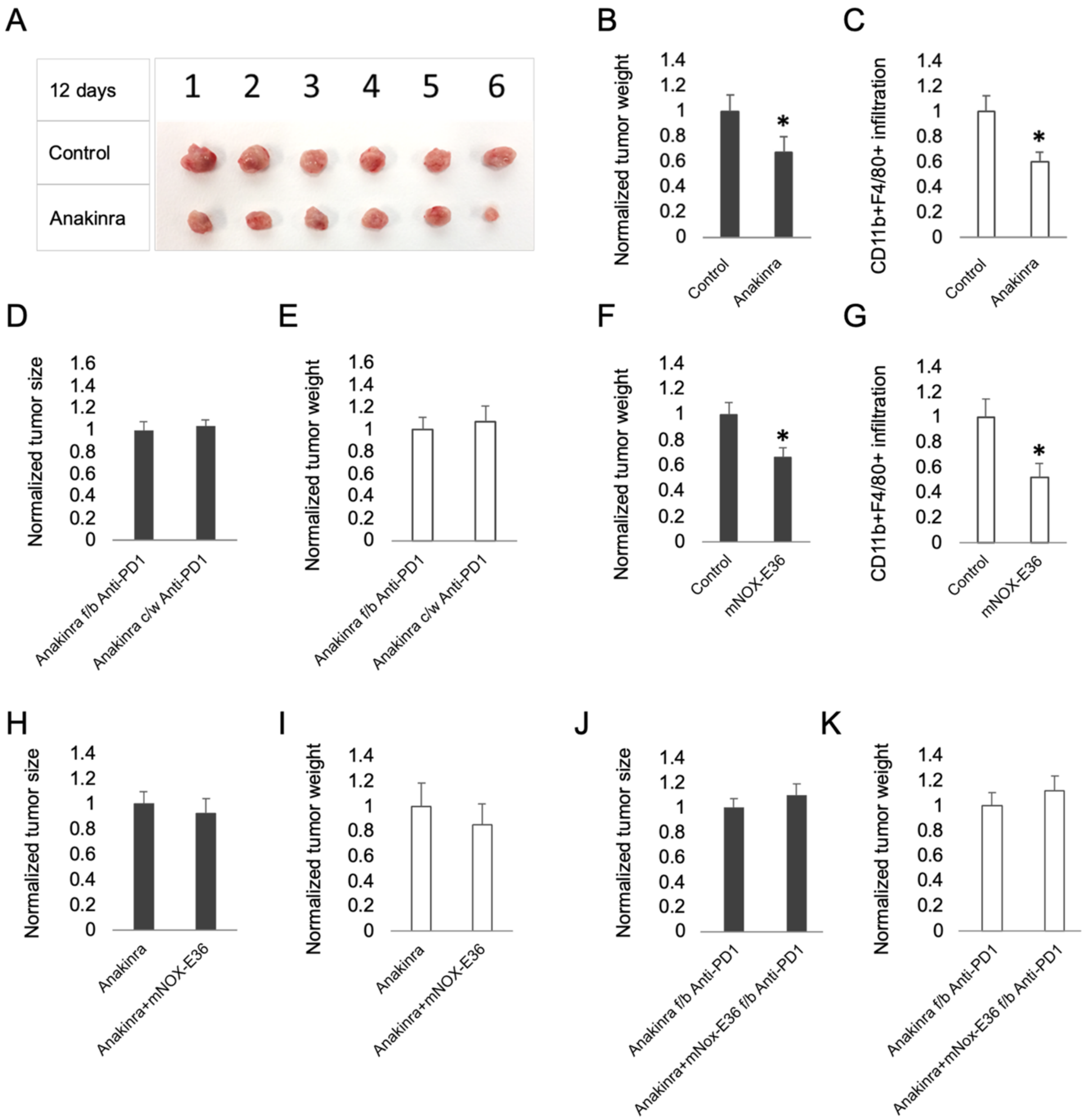

Fig. S3. Inhibition of Notch-regulated cytokines reduces CD11b+F4/80+ TAMs and tumor weight. **A**, Excised tumors following stage 1 control or anakinra treatment. **B**, Tumor weight after stage 1 anakinra (n = 6 tumors/group). **C**, Flow cytometric analysis of CD11b+F4/80+ TAMs after stage 1 anakinra (n = 6 tumors/group). Tumor size (**D**) and weight (**E**) following anakinra f/b anti-PD1, or anakinra c/w anti-PD1 (anakinra for stages 1 and 2 and anti-PD1 for stage 2) (n ≥ 7 tumors/group). **F**, Tumor weight after stage 1 mNOX-E36 treatment (n ≥ 7 tumors/group). **G**, Flow cytometric analysis of

CD11b+F4/80+ TAMs after stage 1 mNOX-E36 treatment (n ≥ 7 tumors/group). Tumor size (**H**) and weight (**I**) following stage 1 anakinra or anakinra combined with mNox-E36 treatments (n = 8 tumor samples/group). Tumor size (**J**) and weight (**K**) following stage 2 anakinra or anakinra combined with mNox-E36 followed by anti-PD1 treatments (n ≥ 7 tumor samples/group). Data are presented as mean + SEM. *, $P < 0.05$.

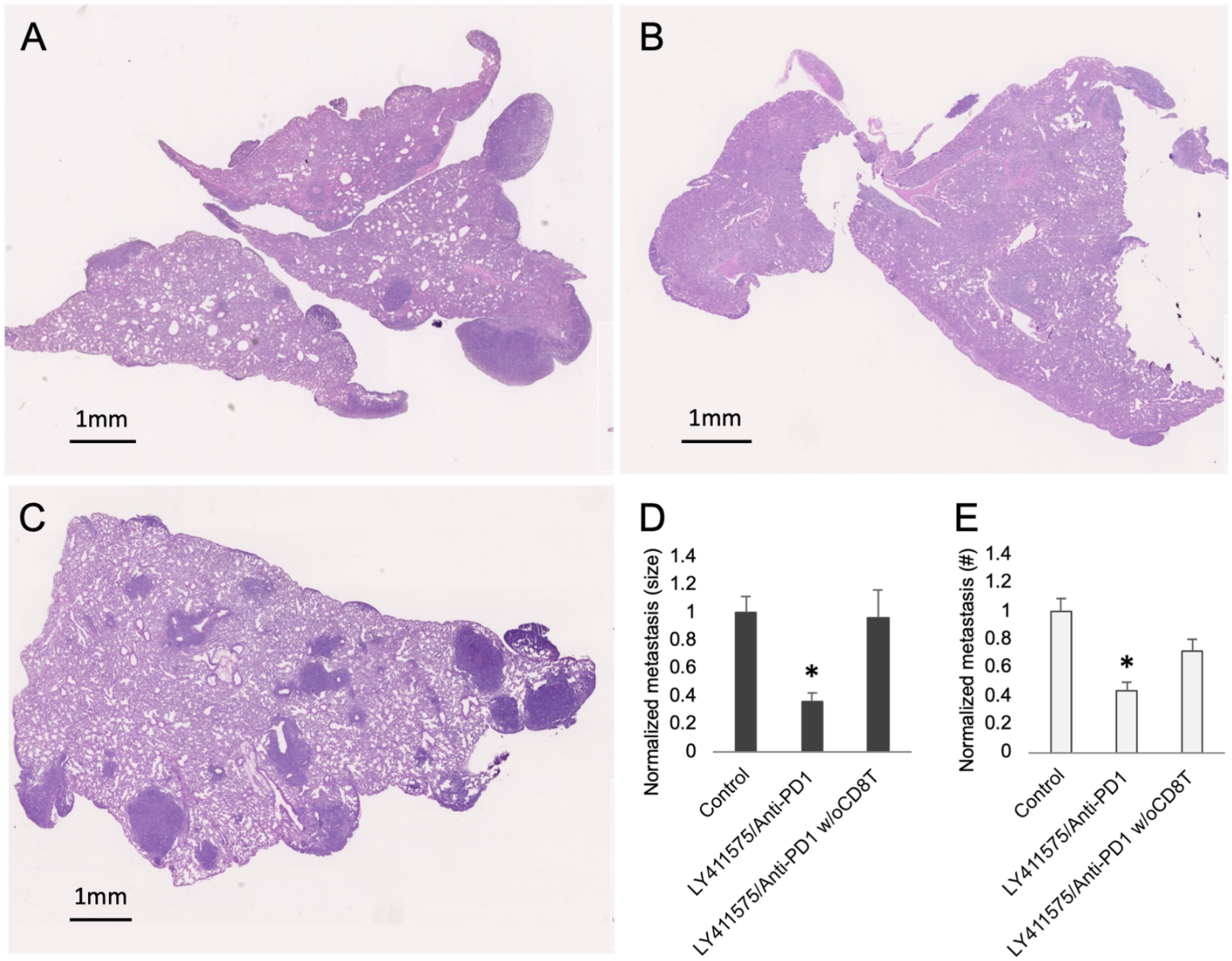

Fig. S4. CD8+ T cells mediate the response of metastases to sequential Notch and immune checkpoint inhibition Representative histological images of metastatic foci in lungs following stage 2 treatment with (**A**) vehicle control and (**B**) LY411575 f/b anti-PD1 in BALB/c mice, or (**C**) LY411575 f/b anti-PD1 in BALB/c mice in which CD8+ T cells were depleted. Normalized size (**D**) and number (**E**) of metastases following stage 2 treatment (n ≥ 6 mice/group). Data are presented as mean + SEM. *, $P < 0.05$.

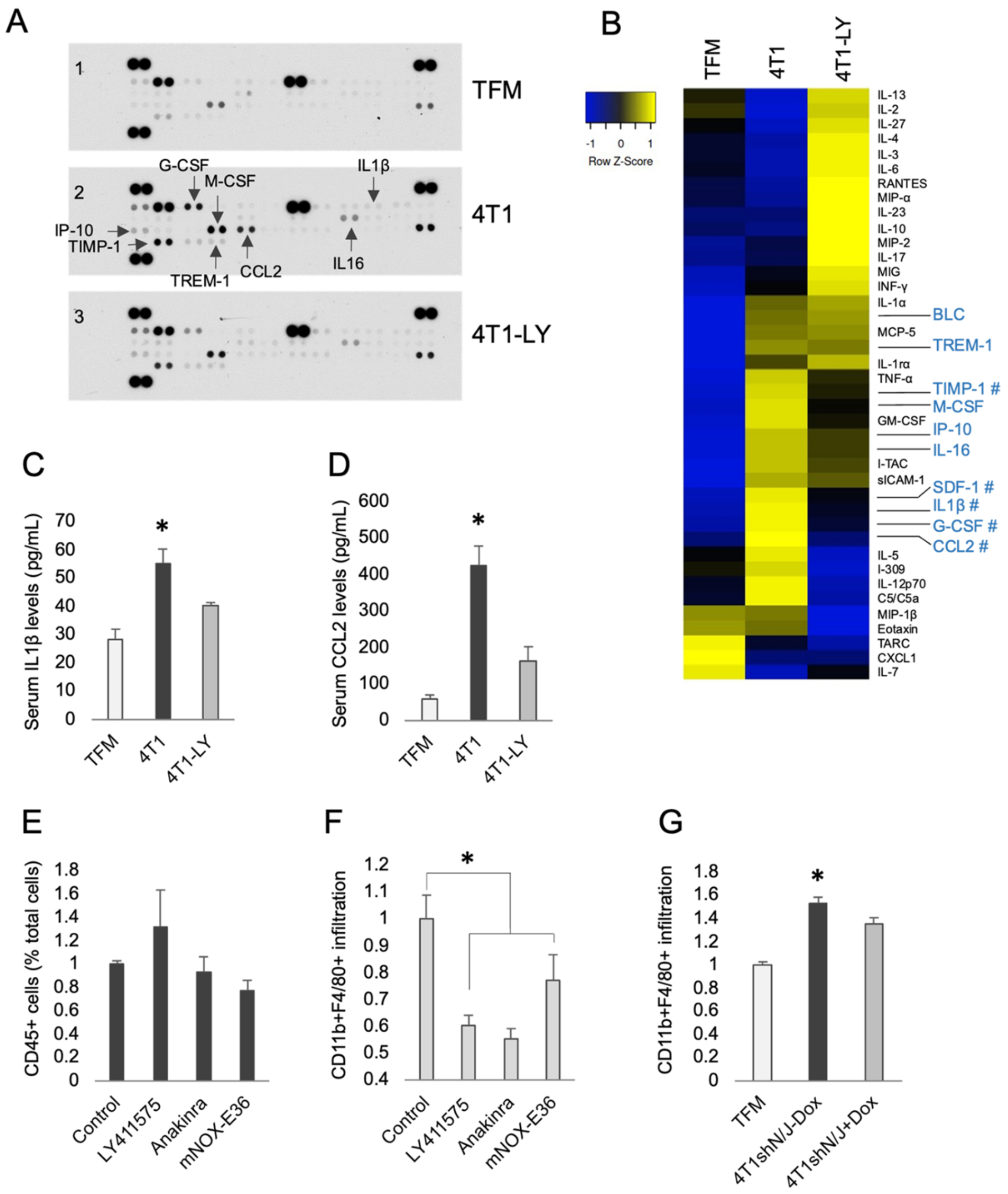

Fig. S5. A, Murine cytokine arrays probed with serum from tumor-free BALB/c mice (TFM), stage 1 vehicle control-treated mice bearing 4T1 allografts (4T1), or LY411575-treated mice bearing 4T1 allografts (4T1-LY). **B**, Heat map of relative cytokine level measured by array densitometry. The color key relates the heat map colors to the standard score (z-score), i.e. the deviation from row mean in units of standard deviations above or below the mean. Cytokines increased >2 fold in 4T1 compared to TFM serum are shown in blue.

# indicates cytokines decreased more than 30% in 4T1-LY. Serum levels of IL1β (**C**) and CCL2 (**D**) by ELISA (n = 3 mice/group). Flow cytometric analysis of CD45+ cells (**E**) or CD11b+F4/80+ TAMs (**F**) in lung after stage-1 treatment with control, LY411575, anakinra, or mNox-E36 (n ≥ 3 mice/group). **G**, Flow cytometric analysis of CD11b+F4/80+ TAMs in lung of TFM or mice bearing 4T1shN/J allografts treated without (-) or with (+) doxycycline (Dox) (n = 9 mice/group). Data are presented as mean + SEM. *, $P < 0.05$.

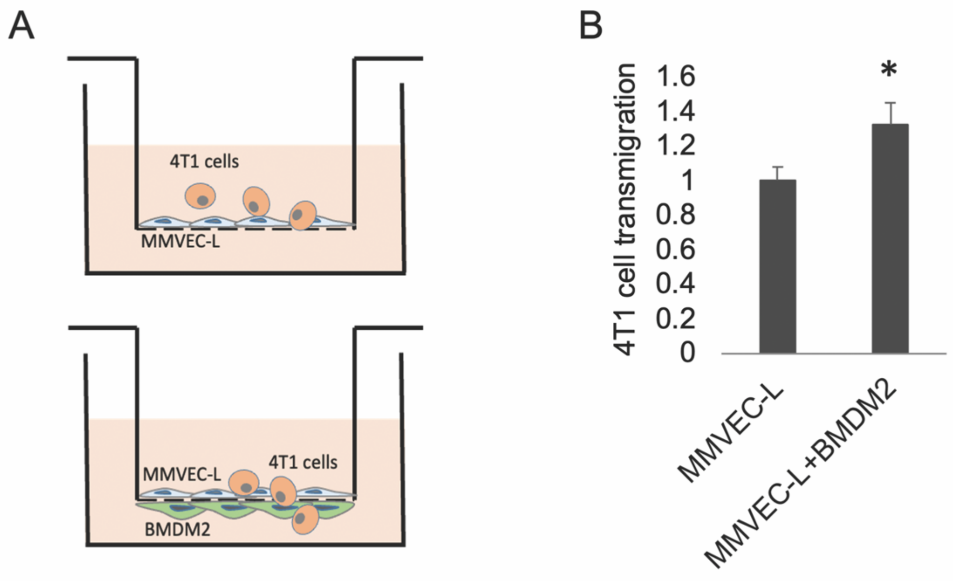

Fig. S6. Bone marrow-derived M2-like macrophages promote extravasation of 4T1 cells through microvascular endothelium. **A**, Schematic diagram of the *in vitro* tumor cell extravasation model, without or with the presence of BMDM2 – see materials and methods. **B**, Quantification of relative 4T1 extravasation without or with the presence of BMDM2 (n = 6/group). Data are presented as mean + SEM. *, $P < 0.05$.
